# Place Work on a Scale: What Do We Know About the Association Between Employment Status and Weight Loss Outcomes After Bariatric Surgery?

**DOI:** 10.1007/s11695-021-05388-9

**Published:** 2021-05-21

**Authors:** Marleen M. Romeijn, Marlies Bongers, Daniëlle D.B. Holthuijsen, Loes Janssen, François M.H. van Dielen, Han J.R. Anema, Wouter K.G. Leclercq

**Affiliations:** 1 Department of Surgery, Máxima Medical Center, Veldhoven, The Netherlands; 2 Research School NUTRIM, Department of Surgery, Maastricht University Medical Center, Maastricht, the Netherlands; 3 SGBO, Department of Primary and Community Care, Radboud University Medical Center, Nijmegen, The Netherlands; 4 ArboNed Occupational Health Service, Utrecht, The Netherlands; 5 Department of Public and Occupational Health, Amsterdam Public Health Research, VU University Medical Center, Amsterdam, The Netherlands

**Keywords:** Bariatric surgery, Metabolic surgery, Employment status, Occupational status

## Abstract

Despite the initial successful weight loss after bariatric surgery, a significant amount of patients experience weight loss failure and weight regain. Several factors are known to contribute to this, though the impact of employment status is unknown. The objective of this systematic review was to examine the impact of employment status on post-surgical weight loss outcomes. Eight studies were included with a follow-up ranging between 2 and 10 years. Employed patients seemed to present more weight loss (9.0–11.0% EWL, 1.3–1.6% BMI loss) compared to unemployed patients, but none of these numbers were statistically significant. Moreover, there were contrasting findings in terms of weight regain. This review may highlight the importance of working status after bariatric surgery and warrants further investigation on this topic.

**Supplementary Information:**

The online version contains supplementary material available at 10.1007/s11695-021-05388-9.

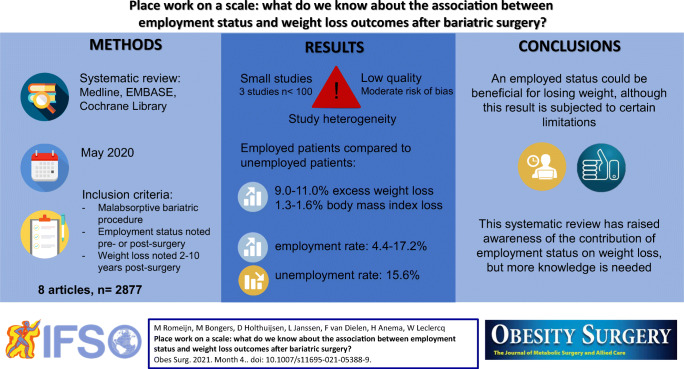

## Introduction

Bariatric surgery has a pivotal role in the treatment of morbid obesity as it effectively reduces weight and obesity related comorbidities [1, 2]. It has a positive effect on physical functioning, psychological health and employment rate [3–6]. Based on a previous systematic review, employment rate has increased by 20% and 16–37% of unemployed patients succeed in finding a job post-surgery (re-employment rate) [5]. Bariatric surgery has also shown to decrease the rate of absenteeism and presenteeism which is the problem of employees being absent, and being present but not fully functioning because of a medical condition [5].

**Fig. 1:**
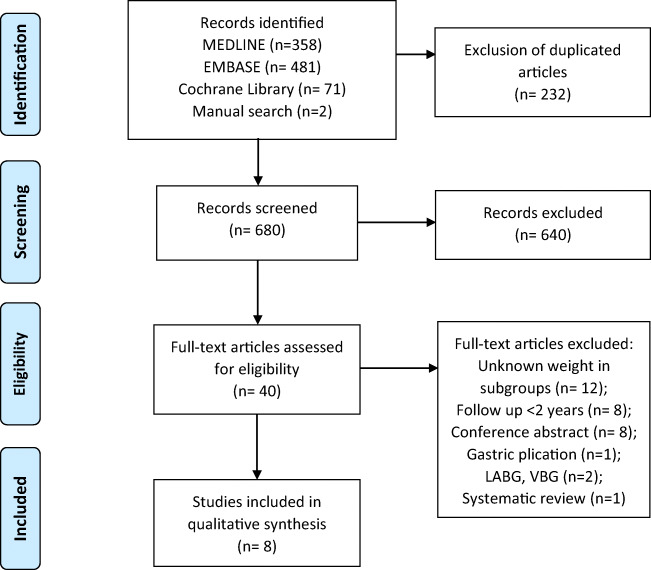
PRISMA flow diagram for study selection. LABG, laparoscopic adjustable gastric banding; VBG, vertical banded gastroplasty

**Table 1:** Study characteristics

Author and year	Country	Study design	Number of subjects (female gender)	Age of subjects^1^	Surgical procedure	Follow-up (years)
Courtney et al. 2018 [26]	UK	Retrospective cohort	1011 (762)	47 (18–78)	Laparoscopic, multiple bariatric techniques^2^	2
Mancini et al. 2018 [27]	France	Retrospective cohort	238 (195)	40 (34–48)	Laparoscopic RYGB (64.7%) SG (35.3%)	2
Jambhekar et al. 2018 [28]	USA	Prospective cohort	713 (622)	41.7 ± 11.2	Laparoscopic SG	2
Keith et al. 2018 [29]	USA	Retrospective cohort	586 (461)	43 (36–51)	Laparoscopic RYGB	9
Hanvold et al. 2015 [30]	Norway	Randomized lifestyle inter-vention study	165 (123)	44 ± 8.6	Laparoscopic RYGB	2
Reid et al. 2018 [31]	Canada	Prospective cohort	48 (36)	50.7 ± 9.4	Laparoscopic RYGB^3^	10
Velcu et al. 2005 [32]	USA	Retrospective cohort	41 (36)	32.4 ± 3.6	Open RYGB	5
Diaz- Guerra et al. 2005 [33]	Spain	Prospective cohort	75 (53)	39	Open BPD of Larrad	5

Abbreviations *BMI* Body Mass Index, *BPD* biliopancreatic diversion, *RYGB* Roux-en-Y gastric bypass, *SG* Sleeve Gastrectomy, *UK* United Kingdom, *USA* United States of America

^1^Expressed in mean with standard deviation or mean with range

^2^Included RYGB, SG, one-anastomosis gastric bypass and gastric banding

^3^Majority of patients were done laparoscopically (±75%)

**Table 2:** Changes in employment status and weight after bariatric surgery

Author	Employment status pre-surgery	Employment status post-surgery	BMI (kg/m^2^) pre-surgery	Weight loss post-surgery based on pre-surgical assessed employment status	Weight regain post-surgery
Courtney et al. [26]	E: 444/746 (59.5%) U: 273/746 (36.6%) Retired: 29/746 (3.9%)	E: 707/1011 (69.9%)^1^ U: 212/1011 (21.0%)^1^ Retired: 92/1011 (9.1%)^1^ 43% was documented <6 months, 60% 7–18 months, 41% 19–30 months post-surgery	E pre: 43 (30–68) U pre: 44 (28–72) Retired pre: 44 (34–54)	E pre: EWL 66% (6–169) U pre: EWL 55% (−159–122) E post: EWL 65% (−7–169)^2^ U post: EWL 56% (−159–159)^2^ Weight loss was measured 2 years post-surgery	Unknown
Mancini et al. [27]	E: 158/238 (66.4%) Disabled and retired patients for 2 years were excluded.	E: 199/238 (83.6%)^3^ Documented 2 years post-surgery	44.9^^^ (41–50)	U pre (*n* = 80): BMI 31.9 ± 6.7 (= 28.9% ∆BMI), EWL 70.8% ± 28.2 Weight loss was measured 2 years post-surgery	Unknown
Jambhekar et al. [28]	E: 300/713 (42.1%) U: 98/713 (13.7%) Retired: 16/713 (2.2%) Disabled: 34/713 (4.8%) Students: 23/713 (3.2%)	Unknown	E pre: 46.0 ± 5.8 U pre: 45.7 ± 6 Retired pre: 43.4 ± 5.2 Disabled pre: 46.4 ± 6.4 Students pre: 47.2 ± 4.9	E pre: 32.4 kg ±13.4 U pre: 33.5 kg ±14.3 Retired pre: 19.7 kg ±7.9 Disabled pre: 21.5 kg ±6.7 Students pre: 49.0 kg ± unknown Weight loss is based on lowest weight noted ≤2 years post-surgery	E pre: 3.8 kg U pre: 5.4 kg Retired pre: 7.9 kg Disabled pre: 0.6 kg Students pre: 3.5 kg Weight noted 2 years post-surgery minus lowest weight
Keith et al. [29]	E: 468/586 (80.0%) U: 43/586 (7.0%) Retired: 26/586 (4.4%), Disabled: 34/586 (5.8%), Student: 14/586 (2.3%)	Unknown	48.0 (44–54)	Unknown	Classified as >15% weight regain 1 year post-surgery E pre: 99/468 (21.2%) U pre: 11/43 (25.6%), Retired pre: 1/26 (3.9%) Disabled pre: 7/34 (20.6%) Student pre: 4/14 (28.6%)
Hanvold et al. [30]	E or student: 101/162 (62.3%) U: 61/162 (37.7%)	E or student: 109/162 (66.7%) U: 54/162 (33.3%) Documented 2 years post-surgery	44.3 ± 5.1	U pre: 9/28 (32.1%) <50% EWL, 45/134 (33.6%) ≥50% EWL U pre: 25/81 (31.3%) BMI ≥30, 29/84 (35.4%) BMI <30 Weight loss was measured 2 years post-surgery	Unknown
Author	Employment status pre-surgery	Employment status post-surgery	BMI pre-surgery	Weight loss post-surgery based on post-surgical or unknown assessed employment status	Weight regain post-surgery
Reid et al. [31]	Unknown	E: 19/48 (39.6%) U: 29/48 (60.4%) Disabled: 5/29 (17.2%) Retired: 6/29 (20.7%) Timing of documentation is unknown	E post: 55.0 ± 14.6 U post: 50.2 ± 12.1	E post: nadir BMI 30.1 ± 9.6 (= 45.3% ∆BMI), EWL 68.6% ±25.2 U post: nadir BMI 28.1 ± 8.0 (= 44.0% ∆BMI), EWL 78.9% ±48.6 Timing of measurement weight loss is unknown	E post: BMI 36.3 ± 10.9 (= 17.1% ∆BMI). Weight regain was measured 9 ± 3 years post-surgery U post-op: BMI 33.2 ± 9.5 (= 15.4% ∆BMI). Weight regain was measured 10 ± 3 years post-surgery
Velcu et al. [32]	E: 14/41 (34.1%) U: 27/41 (65.8%)	E: 16/41 (39.0%) 1 year post-surgery, 18/41 (43.9%) 5 years post-surgery U: 25/41 (60.9%) 1 year post-surgery, 23/41 (56.1%) 5 years post-surgery	E pre: 51.1 ± 5.6 U pre: 55.7 ± 8.3	E (pre or post is unknown): BMI 28.6 ± 3.8 (= 44.0% ∆BMI) U (pre or post is unknown): BMI 32.1 ± 5.9 (= 42.4% ∆BMI) Weight loss was measured 3 years post-surgery	E (pre or post is unknown): BMI 30.1 ± 5.5 (= 5.0% ∆BMI). U (pre or post is unknown): BMI 32.5 ± 5.5 (= 1.2% ∆BMI). Weight regain was measured 5 years post-surgery
Diaz- Guerra et al. [33]	Unknown	Unknown	53.2 ± 10	U or housewife (pre or post is unknown): 6/9 (66.6%) <50% EWL, 28/66 (42.4%) ≥50% EWL^4^ Weight loss was measured 5 years post-surgery	Unknown

Abbreviations: *E* employed, *U* unemployed, *BMI* Body Mass Index, *EWL* excess weight loss, *post* employment status is based on post-surgical assessment, *pre* employment status is based on pre-surgical assessment

Data is expressed in mean, unless otherwise stated in median(^^^)

1 Significant differences in rate of employment, unemployment and retirement between pre- and post-operative (*p* < 0.05)

2 Significant difference in %EWL between employed and unemployed patients post-op (*p* < 0.05)

3 Significant difference in rate of employment between pre- and post-operative (*p* < 0.0001)

4 Significant difference in the amount of unemployed patients with <50% EWL and ≥ 50% EWL (*p* < 0.01)

**Table 3:** Assessment of risk of bias using the ‘Quality Assessment in Prognostic Studies’ (QUIPS) tool

Author	Study participation	Study attrition	Prognostic factor measurement	Outcome measurement	Study confounding	Statistical analysis and reporting	Overall
Courtney et al. 2018 [26]	Moderate	Low	Moderate	Low	High	Moderate	Moderate
Mancini et al. 2018 [27]	Low	Low	Low	Low	Moderate	Low	Low
Jambhekar et al. 2018 [28]	Low	Moderate	Moderate	Moderate	Moderate	Low	Moderate
Keith et al. 2018 [29]	Low	Moderate	Moderate	Moderate	Moderate	Low	Moderate
Hanvold et al. 2015 [30]	Low	Low	Low	Low	Moderate	Low	Low
Reid et al. 2018 [31]	Low	Low	Low	Low	Low	Low	Low
Velcu et al. 2005 [32]	Low	Low	Low	Low	Moderate	Low	Low
Diaz- Guerra et al. 2005 [33]	Moderate	Low	Moderate	Low	Moderate	Moderate	Moderate

*Low* low risk of bias, *Moderate* moderate risk of bias, *High* high risk of bias

Level of risk of bias was determined by judgment of the prompting items belonging to each assessed domain

Non-response refers to the condition when a patient experiences insufficient weight loss, or regains a significant amount of weight [7]. The latter is seen in approximately 20–30% of patients and may result in the return of obesity related comorbidities and a decreased quality of life [8–10]. The etiology of non-response is multifactorial and includes factors like psychological health and compliance with dietary and exercise regimes [11]. In addition to these factors, it is known that pre-surgical BMI, age, type of surgery (e.g. adjustable gastric banding) and anatomical alterations (e.g. pouch and stoma size) are associated with non-response [9, 11]. It is unknown if and how employment status contributes to the development of non-response. Despite this, it is well known that unemployment has a negative effect on both physical and mental health [12].

The underlying principle that may drive the relation between work and post-surgical weight loss can be found in the interaction between employment status and lifestyle behavior. Unemployed patients may experience more psychological stress and depression, potentially leading to decreased physical activity and increased caloric consumption [13–15]. Patients who work in shifts tend to have poorer sleep quality and poorer dietary patterns compared to non-shift workers [16]. Certain workstyle and lifestyle behavior may have predisposed the development of chronic illnesses like morbid obesity in the first place and hypothetically, it may counteract weight loss after bariatric surgery [16, 17].

In order to maximize or maintain post-surgical weight loss, an understanding of the impact of factors like employment status on weight loss outcomes is essential. Up to now, articles primarily described the impact of bariatric surgery on post-surgical employment rate [5, 6], while fewer articles described the predictive value of pre-surgical employment status on weight loss outcomes. Andersen et al. demonstrated that pre-surgical unemployment was a significant predictor for lower %excess body mass index loss (EBMIL) in women two years after sleeve gastrectomy (SG) [18]. Additionally, Cadena-Obando et al. found that lacking a fulltime job pre-surgery was a negative predictor for achieving successful weight loss (≥50%excess body weight loss) one year after various bariatric procedures [19]. Only the study by Stenberg et al. reported long-term results and these results are in contrast to the abovementioned studies, as the authors found that pre-surgical employment as a professional or technician is independently associated with a lower %total weight loss (TWL) five years after Roux-en-Y gastric bypass (RYGB) [20].

A common observation is that an employed status is associated with better weight loss outcomes [18, 19], though the opposite has also been described [20]. A systematic review comparing long-term outcomes in unemployed and employed patients is lacking and therefore, the objective of this study was to systematically review the literature available on employment status of patients that underwent revisional surgery and their weight loss outcomes.

## Methods

This review complies with the recommendations of the Cochrane Handbook for Systematic Reviews and Interventions [21], and was recorded according to the PRISMA systematic review guidelines [22].

### Eligibility Criteria

This review included observational studies and randomized controlled trials (RCTs). Studies were considered eligible if they included patients with a Body Mass Index (BMI) ≥35 kg/m^2^ who had undergone a malabsorptive bariatric procedure (RYGB, SG and biliopancreatic diversion); if they noted employment status pre-surgery or post-surgery, and if they noted change in weight within two to ten years post-surgery. The latter time points were chosen because weight loss reaches its maximum two years after surgery, and weight regain generally occurs in the subsequent years [23]. There were no restrictions regarding the expression of weight, such as change in kg, change in BMI or Excess Weight Loss (EWL). Due to assumed heterogeneity and a lack of information, it was not attempted to further define employment and unemployment. Studies were excluded in case of a restrictive bariatric procedure like adjustable gastric banding and vertical banded gastroplasty because these procedures are not recommended anymore and have little relevance to today’s practice [24]. Besides this, studies were excluded in case of endoscopic procedures like gastric plication. Articles that were designed as animal studies, systematic reviews, letter to the editor and conference abstracts were excluded as well.

### Systematic Literature Search Methodology

The systematic search was conducted on May, 2020. The search was conducted in three electronic databases: MEDLINE (new version 2020), EMBASE and The Cochrane Library. There was no restriction regarding publication date. Keywords in the search strategy included [employment] and [bariatric surgery], and their synonyms. The full search strategies for all databases can be found in supplementary table 1. References within the included articles were screened to retrieve articles that might have been missed.

### Study Selection

RefWorks software was used to manage references and support identification of duplicates. Titles and abstracts were screened on relevance. Full texts were obtained for clarification of eligibility criteria. Reasons for the exclusion of studies were recorded**.**

### Data Extraction

Data extraction was performed in duplicate by two researchers (MR and DH) and was cross-checked by a third reviewer (LJ). The following study characteristics were extracted from the included studies using predefined forms: authors’ names, publication year, country, study design, sample size, type of procedure, gender, mean age, mean weight or BMI or EWL, and employment status. In case of missing data, the author of the article was contacted. It was noted whether the employment status was assessed before or after the assessment of weight loss.

### Outcome Parameters

The primary outcome was the difference in weight loss, and subsequent weight regain, between employed and unemployed patients two to ten years after bariatric surgery. When describing these outcomes, the classification of employment status was preferably based on a pre-surgical assessment as this illustrates the direct impact of employment status on weight loss outcomes. If possible, weight loss outcomes were also described for students, retired and disabled patients. Mean differences in weight or BMI were calculated and if possible, standard deviations were extracted. If possible, the percentage of BMI was calculated and the delta (∆) BMI was extracted. The formula for calculating ∆%BMI from pre-surgical to post-surgical was (pre-surgical BMI – post-surgical BMI)/ (pre-surgical BMI) ×100%. The following formula was furthermore used for the assessment of weight regain: (post-surgical highest BMI– post-surgical lowest BMI)/ (post-surgical highest BMI) ×100%. The advantage of this measurement is that it corrects for baseline differences in BMI, rather than measuring absolute BMI points. The secondary outcome was the difference in (un)employment rate two to ten years after bariatric surgery between pre-surgical employed and unemployed patients.

### Quality Appraisal

In order to assess the methodological quality of the included studies, the Quality in Prognosis Studies (QUIPS) tool was used, as this tool was used. This tool was specifically designed to assess the relationship between the prognostic factor (employment status) and outcome (weight loss and regain) [25]. Two researchers (MR and DH) independently assessed the methodological quality of each study and if consensus could not be reached, inconsistencies were resolved by discussion with a third reviewer (LJ). The following six domains were evaluated: study participations, study attrition, prognostic factor measurement, outcome measurement, study confounding, and statistical analysis and reporting. Each of these domains were eventually rated as low, mediate or high risk of bias.

## Results

The search retrieved 910 bibliographic references and a manual search retrieved two additional articles. A total of 680 articles remained when duplicates were removed. After screening titles and abstracts on relevance, 640 articles were excluded. Full text reading of the 40 remaining articles resulted in the selection of 8 eligible studies. Figure 1 provides a flow diagram of the screening process and inclusion of articles.

### Study Characteristics

Table 1 provides an overview of the included studies. Among the eight included studies, four were retrospective cohort studies [26, 27, 29, 32], three were prospective cohort studies [28, 31, 33] and one study contained baseline data from a randomized interventional study [30]. The studies add up to 2877 participants with a mean follow-up period of 4.6 years ±3.3. The percentage of females ranged between 70.7% and 87.8%. The study of Courtney et al. included patients with Roux-en-Y gastric bypass (RYGB), SG, one-anastomosis gastric bypass, as well as gastric banding [26]. The exact amount of patients undergoing each type of procedure is unknown. Two studies specifically mentioned that the procedure was done laparoscopy [28, 29], while after contacting the corresponding authors four additional studies appeared to include laparoscopic procedures varying in a rate of 100–75% [26, 27, 29, 30].

As shown in Table 2, six studies noted employment status pre-surgery and five studies noted this post-surgery. From these six studies, five studies based their classification of employment status when describing weight loss outcomes, on the pre-surgical assessment [26–30]. In the other three included studies is it unknown whether the employment status used in the description of weight loss outcomes is assessed prior or after to the assessment of weight loss [31–33]. Four studies used self-report questionnaires for the evaluation of employment status [26, 27, 29, 33], while patient files were also commonly used [26, 28, 29]. Three studies described the rate of retired and/or disabled patients separately [26, 28, 29]. Definitions of employment and unemployment were given in only two studies. Mancini et al. classified employed as full-time employed including students and maternity leave [27]; unemployed was classified as part-time employed, temporary impairment and job seeking. Reid et al. described employed and unemployed when this lasted for a minimum of one year. Additionally, unemployed also included retired and disabled participants [31].

### Quality of the Studies (Risk of Bias)

Results for risk of bias were retrieved using the QUIPS tool as shown in Table 3. Overall, four studies were judged as “moderate” risk of bias [26, 28, 29, 33] and four studies were judged as “low” risk of bias [27, 30–32]. Due to a lost to follow-up of 39% after one year [28] and 50% after two years [29], two studies were judged as having a “moderate” risk of attrition bias. Furthermore, four studies were considered to have a “moderate” risk of bias concerning prognostic factor measurement, due to the lack of a questionnaire when evaluating employment status [26, 28, 29, 33]. An important source of confounding was based on the finding that unemployed patients experienced more comorbidities [26] and used more psychopharmaceutical drugs [30].

### Weight Loss Outcomes

Based on the studies that expressed weight loss in %EWL, employed patients lost 66.0% (pre-surgical assessed), 65.0% (post-surgical assessed) and 68.6% (post-surgical assessed) [26, 31]. Additionally, unemployed patients lost 55.0% (pre-surgical assessed), 70.8% (pre-surgical assessed), 56.0% (post-surgical assessed) and 78.9% (post-surgical assessed) [26, 27, 31]. This indicates a difference of 11.0% EWL in favor of pre-surgical employed [26], 9.0% EWL in favor of post-surgical employed [26] and 10.3% EWL in favor of post-surgical unemployed patients [31]. In addition, two studies used cut-off scores of 50% EWL to define success and failure [30, 33]. These studies found that, in patients with successful weight loss, the rate of unemployment ranged between 33.6–42.4%; additionally, in patients with not successful weight loss, the rate unemployment ranged between 32.1–66.6% [30, 33]. These rates were not described for employed patients.

Based on the studies that used BMI, employed patients lost 22.5 and 24.9 points, while the unemployed patients lost 13.0, 23.6 and 22.1 points [27, 31, 32]. Reid et al. reported a greater BMI loss by post-surgical employed patients (2.8 BMI points), while Velcu et al. reported a greater BMI loss by unemployed patients (1.1 BMI points) [31, 32]. These findings were not statistically significant. When calculating %BMI loss, employed patients lost 1.3% (45.3% vs. 44.0%) and 1.6% (44.0% vs. 42.4%) more compared to unemployed patients [31, 32].

Only one study expressed weight loss in kg which was a maximum of 32.4 kg in pre-surgical employed patients and 33.5 kg in pre-surgical unemployed patients [28]. The authors described that an employed status was almost uniformly associated with more weight loss up to two years post-surgery [28].

### Weight Regain Outcomes

Looking at studies that assessed weight regain and %BMI was extracted, post-surgical employed patients gained 5.0% and 17.1%, while post-surgical unemployed patients gained 1.2% and 15.4% five and nine/ten years after surgery, respectively [31, 32]. When expressed in absolute BMI points, this amounted a difference of 1.1 points between the groups and was not statistically significant. Jambhekar et al. found that pre-surgical unemployed patients gained slightly more weight compared to employed patients (5.4 kg versus 3.8 kg) two years after surgery [28]. Moreover, Keith et al. found that pre-surgical unemployed patients presented 4.4% more weight regain (>15% regain one year post-surgery) compared to employed patients [29]. Logistic regression analysis however, revealed that pre-surgical employment status was of no predictive value on weight regain (odds ratio 1.21, *p* value 0.482) [29].

### Change in Employment Status

The amount of pre-surgical employed patients ranged between 34.1% and 80.0% [26–30, 32], and the amount of pre-surgical unemployed patients ranged between 7.0% and 65.8% [29, 26, 28, 30, 32]. The amount of post-surgical employed patients ranged between 39.6% and 83.6% [26, 27, 30–32], while for the post-surgical unemployed patients this was 21% and 60.9% [26, 30–32].

Four studies assessed employment status pre- and post-surgery, thereby making it possible to detect changes. When focusing on the studies with a two year follow-up, employment rate increased by 4.4% [30], 10.4% [26] and 17.2% [27], while unemployment rate decreased by 15.6% [26]. Two studies found that the increase in employment rate was statistically significant [26, 27], and also one study found that the decrease in unemployment rate was statistically significant [26]. Five years after surgery employment rate increased by 9.8% and the unemployment rate decreased by 9.7%. Nevertheless, this lacked statistical significance [32].

## Discussion

Very little is known about the interplay of socioeconomic factors like employment status and their effect on weight loss after bariatric surgery, and how they interfere with the development of non-response. This systematic review aimed to investigate the impact of employment status on post-bariatric surgical weight loss outcomes. In summary, this study found that employed patients experienced more weight loss (9.0–11.0% EWL [26], 1.3–1.6% BMI [31, 32]) two to three years after surgery compared to unemployed patients; however, these findings are not consistent across the included studies and lacked statistical significance [28, 31]. It can be debated whether these amounts of weight loss have sufficient clinical relevance. Nonetheless, it is well known that more weight loss is associated with better clinical outcomes such as an improved health related quality of life and physical fitness [34, 35].

An obvious finding that emerges from this study is that various measurements were used when expressing weight loss (e.g. kg, %EWL, BMI), making a clear comparison between employed and unemployed patients difficult. The diversity in measurements used, as well as the accuracy of these measurements should be criticized. Lost BMI points and kg are highly dependent on their baseline measurement which may give an under- or overestimation of the actual weight loss. This may have been applicable when comparing weight loss reported by Reid et al. and Velcu et al. where there was a difference in baseline BMI [31, 32]. In order to overcome this, we calculated the percentage of BMI which is a more commonly used measurement in articles describing post-surgical weight loss outcomes [36]. Besides the inaccuracy of absolute numbers, it is well known that %EWL is a suboptimal measurement as this is being influenced too much by common differences in baseline BMI [37, 38]. Percentage TWL has been suggested as the most accurate measurement, though none of the included studies used this measurement.

There are four explanations for the finding that employed patients may experience more weight loss. Firstly, employed patients may be greater committed to health promoting behavior [13, 14], thereby positively affecting eating habits, physical activities and subsequent weight loss. Reid et al. demonstrated that post-surgical employed patients performed 1591 more steps per day compared to unemployed patients [31]. Additionally, Courtney et al. showed greater improvements in functional status of pre-surgical employed patients than unemployed patients (35.7% vs. 29.2%) [26]. Though the direction of causality between functional status/physical activity and weight loss is uncertain, it does implicate the importance of employment in post-bariatric patients. A second explanation is that unemployment is related to a lower socioeconomic status, and a lower socioeconomic status is associated with less post-bariatric weight loss [20, 39]. In detail, inferior weight loss have been described in first-generation immigrants, residents in larger cities, patients with low income and patients who receive social security disability [20, 28, 39]. A third explanation for the aforementioned finding is that employed patients are more likely to be adherent to follow-up appointments after bariatric surgery, and attendance to these appointments is associated with better long-term weight loss outcomes [17, 40, 41]. A fourth explanation may be that employed patients experience more routineness in daily life. Because of this, it may take less effort to adjust a new lifestyle, for example learning new eating patterns. This explanation broadly supports the finding that being employed, either part-time or fulltime is associated with less frequent unhealthy eating compared to the unemployed [17]. The finding from Jambhekar et al. that students experienced more weight loss compared to retired patients may underline this theory as students attend school activities which gives them a certain routineness [28]. Employed patients may also show, as result of long working hours, irregular work schedules and thereby have less daily or weekly routineness [42].

Based on the studies that reported weight regain, employed patients gained 1.7–3.8% more BMI than unemployed patients five to ten years after surgery [31, 32], though the opposite was also found (1.6 kg more regain by unemployed patients) [28]. These results lacked any statistical significance. It is difficult to explain why an employed patient would gain more weight and furthermore, a comparison with other studies is hard as these studies lack a sufficient follow-up period to detect weight regain. This warrants further research to obtain more information about the impact of employment status on losing and maintaining weight post-surgery.

This study found that the employment rate increased by 4.4–17.2%, while the unemployment rate decreased by 15.6% after bariatric surgery. A note of caution is necessary as employment and unemployment rates showed large baseline variety and clarification lacked frequently (e.g. distinction between fulltime and part-time). The improvement in employment rate we found is in line with a previous systematic review which overlapped two studies [5, 30, 32]. The observed increase in employment might be explained in this way: weight loss caused by bariatric surgery results in patients becoming more healthy [34, 35], and patients with a better health condition are more likely to find a job as opposed to jobseekers with a poorer health condition [12].

We acknowledge that this review has an important limitation due to its differences in the assessment of employment status at the moment of describing weight loss outcomes. As far as possible, we presented outcomes based on a pre-surgical assessment of employment status and indicated if this was not the case or uncertain. Despite this, it can be debated whether we are looking at the direct impact of employment status on weight loss outcomes or a reverse relation (i.e., impact of weight loss on employment status). Within this relationship, other variables such as the type of job, type of insurance, level of education and neighborhood status may possible interfere. Unfortunately, these variables were very limitedly described in the included studies, highlighting the need for future studies to concentrate on these variables.

Other limitations of this study can be found in methodological issues. To start, the quality of the studies was limited with four studies being assessed as a moderate risk of bias. Selection bias may have been introduced in two studies as it seemed that highly motivated patients returned to follow-up appointments, thereby affecting the documentation of weight and employment status [26, 28]. Furthermore, three studies lacked self-report questionnaires but referred to routinely collected documentation when evaluating employment status, thereby introducing information bias [26, 28, 29]. Multiple studies faced confounding as unemployed patients suffered from functional impairment, co-morbidities and mental health disorders, contributing to their unemployment [26, 33]. None of the studies sufficiently accounted for potential confounders including age, gender, personality disorders, pre-surgical weight and physical activity, while these factors have consistently been associated with weight loss outcomes. Besides this, information lacked about the job type including shift work and a sedentary job, though both are related to obesity [16]. Lastly, it should be mentioned that different surgical procedures were used (laparoscopic versus open; restrictive versus malabsorptive) and it was not always clear how these procedures were distributed in the study cohort [26].

The question rises how the results of this review can be used in the daily practice. We should first be aware of the bidirectional interaction between employment status and post-bariatric weight loss outcomes. We should concentrate on identifying a patients’ employment status in a pre-surgical setting, for example during screening for bariatric surgery, and subsequently in a post-surgical setting. All patients should be motivated and encouraged by health care professionals in bariatric centers to either become or stay employed. A collaboration with occupational health physicians could be beneficial for advising employed patients how they return to work, and for unemployed patients how they acquire a job. Further research should be done to see if the joint effort with the occupational health department is feasible.

## Conclusion

This systematic review showed that an employed status could be beneficial for losing weight after bariatric surgery, though this finding is subjected to heterogeneity in included studies and a lack of statistical significance. The results may implicate that employed patients should be encouraged by health care professionals to return to work and that unemployed patients should be supported to return to labor market. More knowledge is needed to fully understand the interplay between employment status, job type, socioeconomic factors and weight loss outcomes after bariatric surgery.

## Supplementary Information

ESM 1 (DOCX 27 kb)

## Abbreviations

BMI: body mass index
EBMIL: excess body mass index loss
EWL: excess weight loss
Kg: kilograms
RCT: randomized controlled trials
RYGB: roux-en-y gastric bypass
SG: sleeve gastrectomy
TWL: total weight loss
QUIPS: quality in prognosis studies
