# Supplementary material for: Place Work on a Scale: What Do We Know About the Association Between Employment Status and Weight Loss Outcomes After Bariatric Surgery?
Source: Obes Surg. 2021 May 21;31(8):3822–32. doi: 10.1007/s11695-021-05388-9 (PMC8270822; doi:10.1007/s11695-021-05388-9)
Supplement: Supplementary file 1 — (DOCX 27 kb) [file 11695_2021_5388_MOESM1_ESM.docx]

Supplementary table 1

Search strategy MEDLINE (PubMed)

#1	"Bariatric Surgery"[Mesh]
#2	bariatric surger*[tiab] OR bariatric surgical[tiab] OR metabolic surger*[tiab] OR stomach stapling*[tiab] OR gastric bypass*[tiab] OR gastroileal bypass* [tiab] OR gastric sleeve*[tiab] OR gastroplast*[tiab] OR jejonoileal bypass*[tiab]
#3	(obesity[tiab] OR obese[tiab] OR obesitas[tiab]) AND (surger*[tiab] OR surgical[tiab])
#4	#1 OR #2 OR #3
#5	"Employment"[Mesh]
#6	Employment[tiab] OR employed[tiab] OR occupational status*[tiab] OR unemployment[tiab] OR unemployed[tiab] OR return to work[tiab] OR back to work[tiab]
#7	#5 OR #6
#8	#4 AND #7
#9	"Body Weight"[Mesh] OR weigh*[tiab]
#10	#8 AND #9

Search strategy EMBASE (Ovid)

1. exp bariatric surgery
2. (bariatric surger* or bariatric surgical or bariatric operation* or bariatric procedure* or metabolic surger* or stomach stapling* or gastric bypass* or gastroileal bypass* or gastric sleeve* or gastroplast* or jejonoileal bypass* biliopancreatic bypass* or gastric branding or sleev gastrectom*).ab,kw,ti.
3. (obesity or obese or obesitas).mp. and (surger* or surgical).ab,kw,ti.
4. 1 or 2 or 3
5. exp employment/
6. (employment or employed or unemployment or un-employment or unemployed or non-employed or un-employed or occupational status or employment status or return to work or back to work).ab,kw,ti.
7. exp unemployment/
8. 5 or 6 or 7
9. 4 and 8
10. exp body weight/
11. “weigh*”.ab,kw,ti.

12. 10 or 11

13. 9 and 12

Search strategy Cochrane Library

#1	(bariatric surgery OR bariatric surgical OR metabolic surgery OR stomach stapling OR gastric bypass OR gastroileal bypass OR gastric sleeve OR gastroplasty OR jejonoileal bypass):ti,ab,kw
#2	(obesity OR obese OR obesitas):ti,ab,kw AND (surgery OR surgical):ti,ab,kw
#3	#1 OR #2
#4	(employment OR employed OR occupational status OR unemployment OR unemployed OR return to work OR back to work):ti,ab,kw
#5	(weight):ti,ab,kw
#6	#3 AND #4 AND #5
